# The Architecture of EssB, an Integral Membrane Component of the Type VII Secretion System

**DOI:** 10.1016/j.str.2013.02.007

**Published:** 2013-04-02

**Authors:** Martin Zoltner, David G. Norman, Paul K. Fyfe, Hassane El Mkami, Tracy Palmer, William N. Hunter

**Affiliations:** 1 College of Life Sciences, University of Dundee, Dow Street, Dundee DD1 5EH, UK; 2 School of Physics and Astronomy, University of St. Andrews, St. Andrews FE2 4KM, UK

## Abstract

The membrane-bound EssB is an integral and essential component of the bacterial type VII secretion system that can contribute to pathogenicity. The architecture of *Geobacillus thermodenitrificans* EssB has been investigated by combining crystallographic and EPR spectroscopic methods. The protein forms a dimer that straddles the cytoplasmic membrane. A helical fold is observed for the C-terminal segment, which is positioned on the exterior of the membrane. This segment contributes most to dimer formation. The N-terminal segment displays a structure related to the pseudokinase fold and may contribute to function by recognizing substrates or secretion system partners. The remaining part of EssB may serve as an anchor point for the secretion apparatus, which is embedded in the cytoplasmic membrane with the C-terminal domain protruding out to interact with partner proteins or components of peptidoglycan.

## Graphical abstract

**Graphical Abstract:**

**Figure:**
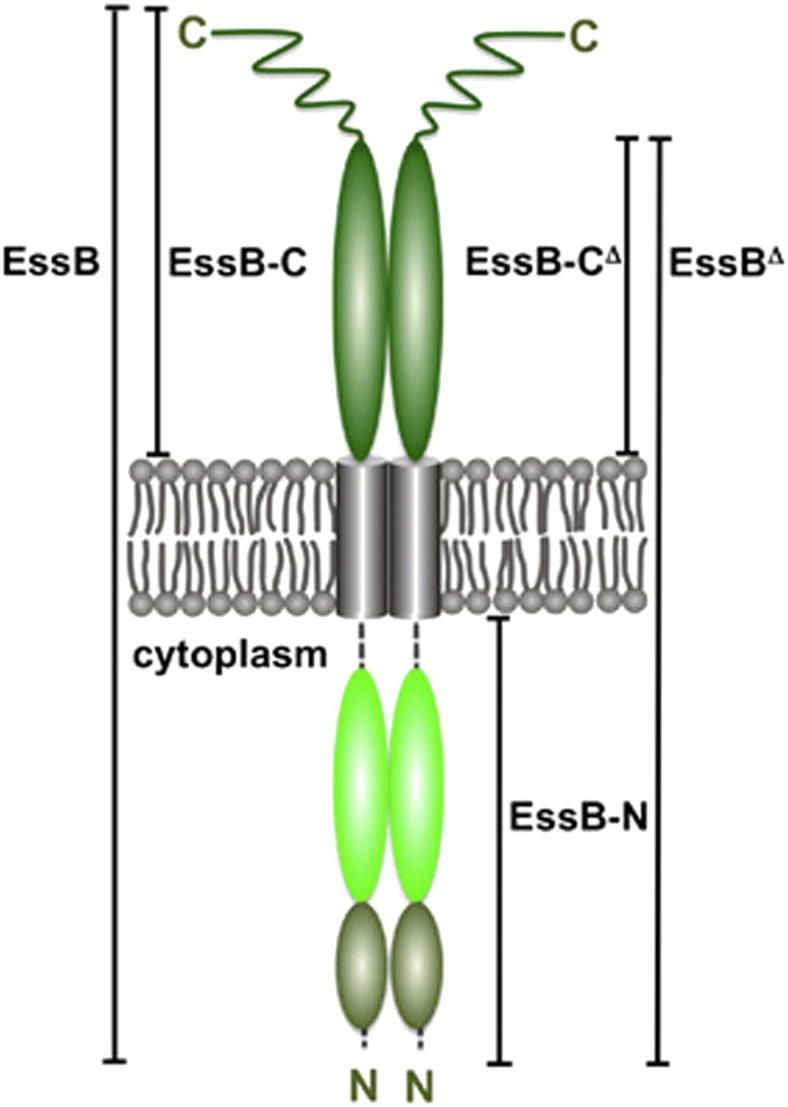

**Highlights:**

► EssB, a membrane-bound component of the type VII secretion system, forms a dimer ► The cytoplasmic segment EssB-N is remarkably similar to pseudokinases ► The extracellular C-terminal domain displays a helical fold ► PELDOR spectroscopy enabled construction of a model of the complete dimer

## Teaser

The architecture of EssB, an essential component of the type VII secretion system, derived from crystallographic and EPR methods by Zoltner et al., reveals a few surprises: a protein that forms a dimer, an extracellular domain with a novel helical fold, and a cytoplasmic segment with remarkable similarity to pseudokinases.

## Introduction

The Type VII or ESX1 secretion system (T7SS, ess) is key to the virulence of important pathogens and to general aspects of gram-positive bacterial fitness (Abdallah et al., 2007). Regarding *Mycobacterium tuberculosis*, the loss of T7SS genes contributes to the attenuated phenotype of the vaccine strain *M. bovis* Bacille Calmette-Guérin (Lewis et al., 2003). *Staphylococcus aureus* also depends on the T7SS to establish persistent infections in a murine pathogenicity model (Burts et al., 2005). Despite the recognized importance of this secretion system to pathogenesis and recent studies to elucidate composition (Houben et al., 2012), there is a paucity of data on the architecture and structure of the T7SS.

T7SS gene clusters are widely distributed in gram-positive bacteria of the phyla Actinobacteria and Firmicutes (Pallen, 2002). These clusters all share the presence of genes encoding ESAT-6 (early secreted antigenic target of 6 kDa) family proteins, the prototypic substrate for the T7SS, and an integral membrane protein called EssC, which possesses FtsK/SpoIIIE-type ATPase domains. All other genes in T7SS genomic loci, inclusive of those reportedly essential for a functional secretion system (Abdallah et al., 2007; Burts et al., 2005), appear to be phylum-specific. At least four genes encode proteins essential for the secretion of the ESAT-6-family proteins in *S. aureus* (Chen et al., 2012). One of these is EssB, a 50-kDa bitopic integral membrane protein that is conserved among T7SS gene clusters in Firmicutes.

**Figure 1:**
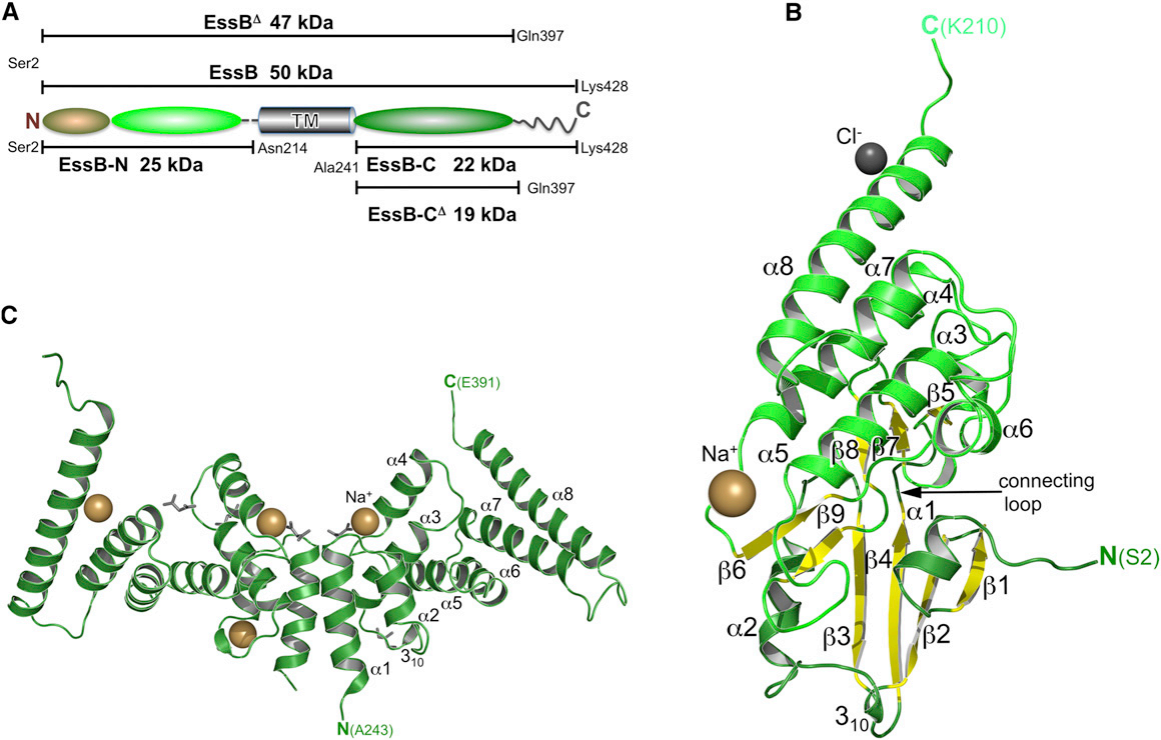
Structures of the Soluble N- and C-Terminal Fragments of EssB (A) Schematic of EssB, predicted domain borders. EssB is a 50-kDa integral membrane protein with a single predicted transmembrane segment (TM, gray cylinder) spanning Ile219 to Phe240. The constructs and the nomenclature used in this study are given. (B) Cartoon representation of EssB-N. Helices are green, and β strands are yellow. A sodium ion (bronze) and a chloride ion (gray) are shown as spheres. The loop connecting the N- and C-terminal domains is identified. (C) Cartoon representation of the extracellular fragment EssB-C^Δ^. Malonate and glycerol are drawn as gray sticks and sodium ions as bronze spheres. Also see Figure S1.

We targeted EssB from the thermophilic gram-positive bacterium *Geobacillus thermodenitrificans* for characterization. Our bioinformatics analyses predict two similar sized segments on either side of the membrane (Zoltner et al., 2013). Membrane-bound EssB and isolated soluble fragments were expressed, purified, and crystallized (Figure 1A). The crystals of detergent-solubilized dimeric EssB^Δ^ were poorly ordered, but structures of the N- and C-terminal soluble fragments, 25 kDa EssB-N and 19 kDa EssB-C^Δ^, were determined by single-wavelength anomalous diffraction (SAD) and refined to 1.7 Å and 2.4 Å resolution, respectively. EssB-N is dimeric in solution but crystallizes as a monomer. It displays a fold related to that of protein kinases, which is composed of two globular domains separated by a cleft (Zoltner et al., 2013). The dimeric EssB-C^Δ^ displays a helical fold that extends over the cytoplasmic membrane, and the structure suggests that this segment contributes significantly to the dimerization of EssB. The topside surface of EssB-C^Δ^, with a cradle-like structure, exhibits large grooves, formed between helical bundles, well matched to interact with helical features of binding partners.

We exploited pulsed electron double resonance (PELDOR) spectroscopy (Milov et al., 1981; Pannier et al., 2000) to obtain distances between pairs of single nitroxide paramagnetic spin labels (Jeschke et al., 2002; Schiemann and Prisner, 2007) in the detergent-solubilized EssB dimer. The crystal structures provided information on positions for label placement, and the PELDOR data, in combination with the X-ray structures, guided construction of a model depicting the overall architecture of this key, membrane-bound component of the T7SS.

## Results and Discussion

### Overview

A recent study of recombinant *S. aureus* EssB reported that the full-length protein was membrane bound but that soluble aggregates formed in the cytoplasm (Chen et al., 2012). The soluble material was investigated; surprisingly, no effort to characterize the membrane-bound protein is described.

We prepared five different constructs and analyzed the encoded polypeptides (Figure 1A). The two membrane-bound polypeptides, EssB and EssB^Δ^, were purified with yields of approximately 1–1.5 mg/l of bacterial culture. The truncation of 31 residues to provide EssB^Δ^ was designed to circumvent disorder at the C terminus that might compromise crystal order (growth). A determined crystallization effort produced crystals of EssB^Δ^, which although of good appearance and size up to 80 μm, only diffracted to 9 Å resolution at a synchrotron microfocus beamline (European Synchrotron Radiation Facility [ESRF], ID23-2). We were unable to index the poor quality diffraction.

Three constructs encoding isolated fragments were prepared. These are EssB-N, the N-terminal fragment with predicted cytoplasmic localization; the C-terminal fragment EssB-C, predicted to reside on the *trans*-side of the membrane; and the C-terminally truncated version EssB-C^Δ^. The yields for each of these were around 10 mg/l of bacterial culture.

We determined the crystal structure of EssB-N using a construct comprising the entire predicted cytoplasmic fragment (Ser2 to Asp214). For the extracytosolic fragment, two structures were determined. First, a low-resolution structure of EssB-C comprising residues Ala241 to Lys428 was obtained and revealed that the C terminus is inherently flexible because it is not resolved in the electron density (discussed later). This polar C-terminal region is divergent, variable in length, or completely absent in orthologs. A higher resolution structure of EssB-C^Δ^, residues Ala241 to Gln397, resulted when the flexible C terminus was omitted from the recombinant polypeptide.

### Structure of EssB-N

The structure of EssB-N was determined at 1.7 Å resolution (Figure 1B; Table 1; Figure S1A available online). Initial phases were derived from a SAD experiment using SeMet-substituted protein. The asymmetric unit consists of a single polypeptide folded into an elongated structure, approximately 55 Å long and 35 Å wide, which consists of two domains. The N-terminal domain is dominated by a four-stranded antiparallel β sheet. The strand β4 leads into a loop connecting the domains. The C-terminal domain consists of a helical bundle formed by two antiparallel helix pairs, α4/α5 and α7/α8. A cleft, approximately 30 Å deep, is formed between the two domains, blocked at one side by the interdomain connecting loop that links β4 to β5. A further interdomain contact is made by a loop interspersed between α2 and β3.

The EssB-N segment resembles the bilobal structure of ATP-dependent protein kinases, i.e., an N-terminal domain or N-lobe with a β sheet packed against an intersecting α-helix, and a predominantly α-helical C-terminal domain (C-lobe; Zoltner et al., 2013). In protein kinases, the cleft formed between the two lobes contains the catalytic machinery and is where ATP and protein substrates bind. The most similar structural EssB-N ortholog identified is the catalytic domain of the mammalian Tyr kinase Abl in an inactive conformation (Protein Data Bank [PDB] code 2G1T; Levinson et al., 2006), which exhibits 9% sequence identity. An overlay provides a Z-score of 10.4 and a root-mean-square deviation (rmsd) of 4.4 Å for an alignment of 173 Cα-atoms. These values suggest a distant evolutionary relationship but the catalytic residues are absent from EssB-N. However, at the C-terminal inner surface of the EssB cleft, as well as at the domain contact area, a remarkable degree of structural similarity is evident (Zoltner et al., 2013). This region of the protein kinase structures is key for substrate recognition and binding (Levinson et al., 2006) and suggests a function for EssB-N as a protein-protein interaction module using the stable modular entity of the protein kinase fold.

EssB-N of *G. thermodenitrificans* and *S. aureus* (Zoltner et al., 2013) share a sequence identity of approximately 20%. A structural alignment (Figure S1C) of 170 Cα-atoms has a Z-score of 16.7 and an rmsd of 2.5 Å. The *G. thermodenitrificans* EssB-N structure is more complete with 209 residues, in contrast to 176 residues in the *S. aureus* protein. The extended model serves to identify an additional strand, β1 of the N-terminal domain β sheet, which is preceded by a short helix, and also to extend the C-terminal helix toward the membrane. These orthologous fragments crystallize as a monomer despite forming stable dimers in solution (discussed later). In both structures, the contacts formed with symmetry-related molecules appear unlikely to support the existence of thermodynamically stable dimers in solution. In addition, there is no combination of molecules with an orientation relevant to membrane association, i.e., with the C-terminal sections directed in a similar way.

To analyze the influence of external conditions on the quaternary structure of EssB-N, we subjected the protein to size exclusion chromatography in different buffer systems. EssB-N elutes as a single species with a molecular weight that corresponds to a dimer in various buffers (see Experimental Procedures), which is consistent with its migration with an apparent mass of 45 kDa in BN-PAGE (Figure S2A). However, in 0.1 M citrate buffer pH 5.5, the buffer used in crystallization, more than 10% of the eluted EssB-N is monomeric (Figure S2B). The transition to a monomeric state appears to be influenced by a combination of low pH and high ionic strength, which matches the crystallization conditions used for EssB-N where we may have been selecting out the monomer. The observed stability of the EssB-N dimer suggests biological relevance. However, conditions in the bacterial cytosol, in terms of ionic strength for example, might allow a conditional association of the cytoplasmic domains in the EssB dimer responsive to the binding of other factors.

### The Extracellular EssB-C

The EssB-C polypeptide gave poorly ordered, highly anisotropic tetragonal crystals. Nevertheless, a low-resolution crystal structure was obtained by SAD methods. The structure indicated that the C terminus was disordered; hence, a truncated protein, EssB-C^Δ^, was targeted with the aim of improving diffraction quality. This proved successful; more ordered monoclinic crystals were obtained. SeMet SAD phasing was repeated and the native structure was refined to 2.4 Å resolution (Table 2).

### Structure of EssB-C^Δ^

EssB-C^Δ^ displays an all-helical architecture not previously described and there is no example of this topology in the PDB. Approximately 75% of residues are in α helices arranged into a series of coiled-coil motifs that are predicted to extend across the cytoplasmic membrane (Figures 1C and S1B). The structure starts at α1, with Ala241 at the transmembrane border, potentially the continuation of the helix that transcends the membrane (Figures 1C and S1B). The antiparallel coiled-coil of α1 and α2 is followed by a loop facing the membrane surface. This loop, carrying a turn of 3_10_-helix, connects to the next antiparallel side-by-side helix pair (α3, α4). At the lower end of α4, a sodium:malonate ion pair is bound. The four remaining helices, α5–α8, form two sets of antiparallel coiled-coils.

Size exclusion chromatography indicated that EssB, EssB-C, and EssB-C^Δ^ form dimers. The asymmetric unit of EssB-C^Δ^ consists of four molecules, which are labeled A to D. An average rmsd value of 1.38 Å is obtained from the least-squares fit of the Cα positions of individual subunits with each other. This relatively high value is strongly influenced by a few large differences, in excess of 10 Å, at the C-terminal segments but overall the molecules are similar. To identify the relevant dimer, we sought an interface common to each of the four molecules with the important consideration that the dimeric association should be compatible with each molecule having the same orientation with respect to the membrane. The relevant dimer is generated by crystallographic symmetry operations −x+1/2, y−1/2, −z+1 and −x, y, −z (AB and DC pairings), respectively. The dimer is primarily formed by interacting residues on α1, α2, and the loop linking α4 to α5. The interaction surface uses only about 7% of the solvent-accessible surface, a low value compared with dimers of similar sized polypeptides. However, with a solvation free energy gain of −14 kcal/M (estimated using PISA; Krissinel and Henrick, 2007), the dimer is predicted to be thermodynamically stable. Moreover, in the full-length protein, the transmembrane helices would be expected to also contribute to dimerization. One of the interface regions formed between molecules in the asymmetric unit has a larger interaction surface area of approximately 12%. However, with a solvation free energy gain of only −5.1 kcal/M, it is predicted to be less stable than the dimer depicted in Figures 1C, S1B, and S2C. The same dimer identified in the monoclinic crystal structure is also evident in the low-resolution tetragonal structure that contains two EssB-C molecules in the asymmetric unit. It is generated by the crystallographic symmetry operation −y−1/2, x−1/2, z−1/4. The assignment of this as the physiologic dimer is further corroborated by PELDOR spectroscopy (see below).

### PELDOR Spectroscopy

We applied PELDOR spectroscopy to measure intermolecular distances in the dimeric, membrane-bound EssB^Δ^ to obtain information about the overall architecture (Table 3; Figure S2D). The structures were inspected for surface-exposed, nonconserved residues within secondary structure elements at which cysteines labeled with MTSSL might be placed without disruption of the structures and that might usefully provide information on orientation. Eight such positions were identified and tested.

**Figure 2:**
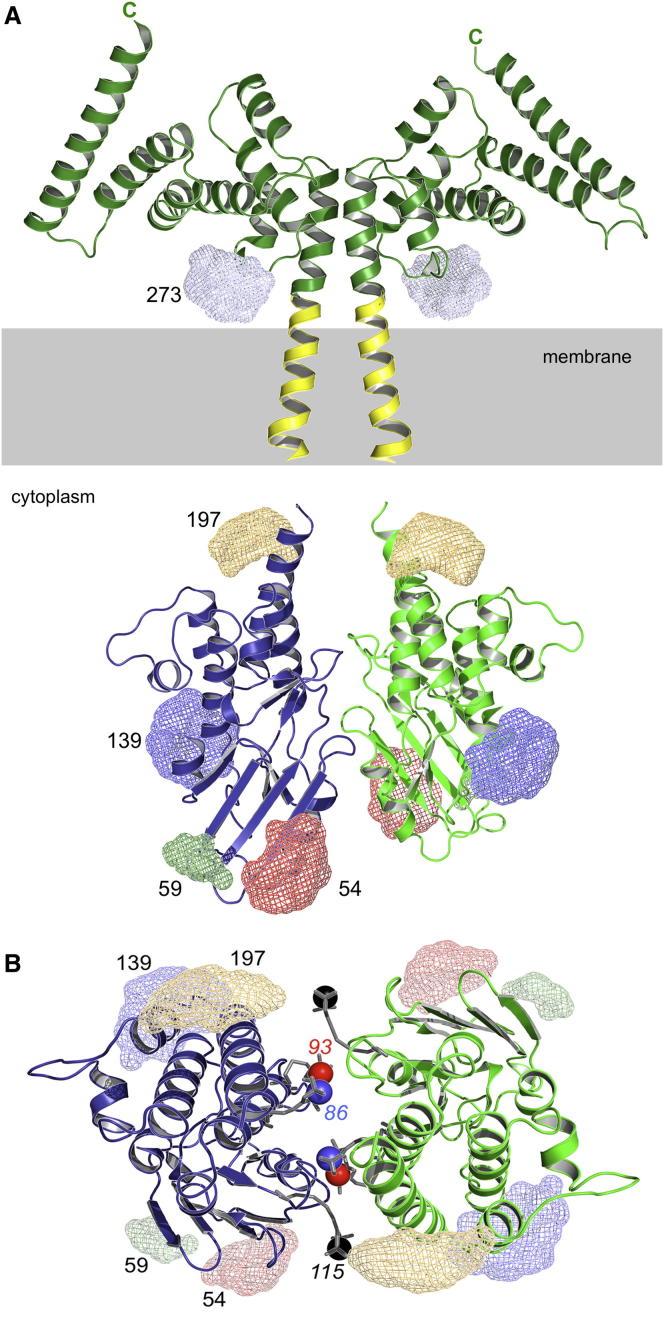
Predicted Architecture of EssB with Spin-Labeling Positions (A) Schematic EssB model in cartoon representation showing positions of MTSSL-labels (see also Movie S1). Ensembles of possible MTSSL nitrogen positions on the five mutant positions are shown as color-coded mesh for clarity (D54C in red, E59C in green, E139C in dark blue, E197C in orange, and E273C in light blue). The model is assembled with the EssB-C dimer and modeled TM-segment (yellow) extended down through the membrane toward the docked cytoplasmic EssB-N fragments. The gray box denotes the cytoplasmic membrane. See also Movie S1. (B) Topview of the EssB-N model. Three additional label positions, producing oscillation free data, are shown in sticks with the paramagnetic amine oxide drawn as color-coded sphere (R86C in blue, S93C in red, and N115C in black). Also see Figure S2.

The three endogenous cysteines were first mutated to serine to prevent labeling at those sites, then eight single-site mutations were constructed, the encoded proteins purified and labeled. One position was in the extracellular domain (Glu273) and the rest in the intracellular domain (Asp54, Glu59, Arg86, Ser93, Asn115, Glu139, and Glu197; Figure 2; Movie S1). These mutated proteins eluted as a single species identical to the wild-type dimer during size exclusion chromatography.

The extracytosolic E273C showed a major distance distribution of 44 Å, compared to the value of 43 Å derived from the EssB-C^Δ^ crystal structure (Figure S3A), essentially confirming the assigned dimer structure (Figures 1C and S1B) and ruling out the physiologic relevance of the crystallographic dimer contained in the asymmetric unit (AD pairing) with a theoretical interspin distance of 49 Å.

The remaining positions were investigated in the context of the whole, detergent-solubilized, EssB^Δ^ dimer (Figure S3B) and two (D54C and S93C) were additionally investigated as the isolated intracellular fragment EssB-N. These two mutants were analyzed by size exclusion chromatography before and after MTSSL-labeling. While D54C eluted solely as a dimer, for S93C a small fraction of EssB-N monomer could be detected after labeling (data not shown). The large MTSSL-group at position 93 appears to destabilize the dimer, a strong indication that Ser93 is indeed located on or near the dimer interface (Figure 2B).

Of the seven intracellular positions investigated, four (D54C, E59C, E139C, and E197C) provided PELDOR data showing clear oscillations (Figure S3C). Position E139C, on α5, exhibits an interspin distance of 58 Å (Figure S3B). The data are comparable to those obtained for the E273C mutant but with a significantly smaller oscillation depth that might indicate partial dimerization, yet under the conditions used in SEC, this sample appeared only to be a dimer. The D54C label, localized on β3, was investigated in both solubilized EssB^Δ^ and EssB-N. The distance distributions are virtually identical with an interspin distance of 55 Å (Figure S3B). PELDOR data from EssB-N show oscillations with a much smaller oscillation depth than the solubilized EssB suggesting that, under these conditions, not all the sample is in a dimeric form. Such an observation is consistent with other data on this isolated fragment. The mutant E59C provided a separation of 66 Å, and E197C, located on the C-terminal α8, provided a distance of 38 Å (Figure S3B).

The PELDOR data derived on the remaining three positions (R86C, S93C, and N115C) were devoid of oscillations (Figure S3D), and Tikhonov regularization did not resolve clear distance distributions. This may have been a consequence of steric restrictions on the spin labels because they occur at the interface (Figure 2B). The PELDOR data are consistent with the dimerization of the membrane-bound protein, and the extra- and intracellular domains. Well-defined distances (Table 3; Figures S2D and S3E) informed the generation of an EssB dimer model by docking the intracellular monomers with restrained, rigid body, molecular dynamics in conjunction with the fixed structure of the extracellular segment EssB-C^Δ^ (Figure 2; Movie S1).

### The Architecture of EssB and Functional Implications

EssB^Δ^ and the extracellular segment, EssB-C^Δ^ are stable homodimers. The PELDOR distance measurements unambiguously confirmed this for the intracellular domain both in the intact, detergent-solubilized and isolated forms. EssB-N appears to be a conditional dimer. Modeling, based on the structures and incorporating PELDOR distance restraints, generated an EssB dimer model consistent with the distance information and with data from labels that appear to disrupt the dimer (Figure 2; Movie S1). To emphasize this last point, the labeling at positions 86, 93, and 115 did not provide distance information to assist with modeling of the dimer. All three mutant/labels appear to destabilize the dimer, and the model locates them at the interface, perfectly positioned to disrupt the association (Figure 2B).

The extracellular segment dimer observed in the structure of EssB-C^Δ^ is retained, as previously detailed, and embedded in the membrane by a predicted transmembrane helix. Based on secondary structure predictions and sequence conservation (Zoltner et al., 2013; Figure S3F), a similar topology and dimer are predicted for orthologs. On the other side of the membrane, a weaker dimeric association is formed by EssB-N. Here, the contact interface uses α2, α4, α8, and three loop regions (Asn115-Pro120, Val95-Leu103, and Glu44-Ile49; Figure S3G). The interaction surface is primarily polar and composed of alternating acidic and basic patches that exhibit a charge complementarity between monomers. The middle section of the EssB-N interface is more hydrophobic in nature and a basic patch is positioned near β6, recessed in a groove.

The N-terminal segment of EssB may not be a major contributor to the stability of the dimeric, intact protein. Interactions involving the transmembrane helices and the C-terminal segment appear to be more important in this context. Nevertheless, sequence alignments and secondary structure prediction indicate that the helical regions and loops that appear to participate in the dimerization of EssB-N are conserved (Figure S3H).

**Figure 3:**
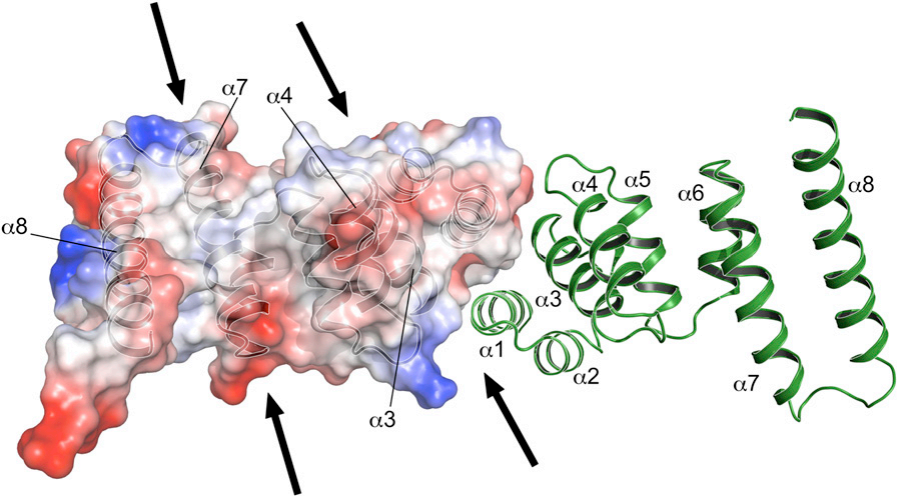
A van der Waals Surface Representation of the EssB-C^Δ^ Dimer Topview of the EssB-C^Δ^ dimer. One subunit is depicted with ribbon format and the other has the van der Waals surface shown and colored according to chemical properties: red, acidic; blue, basic; white, nonpolar. The arrows mark entrances to grooves on the surface of the protein. Also see Figure S3.

Our model and characterization of EssB offers clues to the mode of action as part of the T7SS complex. The C-terminal portion is directed out from the cytoplasmic membrane. If we consider the flexible C-terminal tail, then this may extend approximately 50 Å above the membrane. EssB is then positioned to interact with components of the T7SS, and/or with components of the cell wall. The topside surface exhibits two large grooves, one formed between α4 and α8 and one between the helix pair α3-α4, above the dimer interface (Figure 3). A mixture of polar and nonpolar groups lines the grooves and might facilitate interactions with partners. The C-terminal segment could therefore function like a toggle-bolt to tether and localize EssB and relevant T7SS complexes to the membrane.

The structural similarity of the cytoplasmic segment to Ser/Thr protein kinases, fluxional behavior, and overall topology of EssB is reminiscent of receptor protein kinases such as the eukaryote-like Ser-Thr kinases found in *M. tuberculosis*, PknB, and PknG (Pereira et al., 2011). Ligand binding to an extracellular domain promotes conformational changes resulting in receptor dimerization or stabilization of a loose dimer, which in turn allows *trans*-activation of the cytoplasmic kinase domains with the overall result that a signal transduces the membrane. The fluxional behavior of EssB-N appears to be an intrinsic property that might be exploited by a molecular partner, which could be a substrate of T7SS or a component of the secretion apparatus itself.

It is intriguing that encoded on the T7SS gene cluster is EssC, a large, membrane-bound ATPase. The ATPase activity resides on the C-terminal part of the protein and can provide the motive force for the T7SS. The N-terminal domain of EssC, predicted to reside in the cytoplasm and for which a structure has been determined (Tanaka et al., 2007), displays a combination of two forkhead-associated (FHA) domains. FHA domains are phosphopeptide, in particular phosphothreonine, recognition motifs. They are widely dispersed from prokaryotes to higher eukaryotes and are implicated in intracellular signal transduction, protein transport, and protein degradation (Liang and Van Doren, 2008). Most recently, they have also been implicated in the function of the type III secretion system (McDowell et al., 2011). The FHA domains of EssC may interact with other proteins in the ESX-1 secretion system, with substrates or regulators for example. It is intriguing to find a phosphothreonine-type peptide recognition module in one component of the T7SS and a Ser/Thr kinase protein recognition fold on another component, with both being membrane localized. It will be of interest to confirm if and how these domains interact.

## Experimental Procedures

### Cloning and Mutagenesis

The full-length *G. thermodenitrificans* NG80-2 EssB (Uniprot: A4IKE6) coding sequence (codon optimized for *Escherichia coli* K12 (Genscript)) and EssB^Δ^, a construct encoding a product truncated by 31 C-terminal residues, were cloned into the *Sal1/Xho1* site of a modified pET27b vector (Novagen). Constructs to produce the predicted cytoplasmic fragment EssB-N (Figure 1A) and the two fragments predicted to reside *trans* of the cytoplasmic membrane (Figure 1A), EssB-C (residues 241–428) and EssB-C^Δ^ (residues 241–397), were generated accordingly. All primers are listed in Table S1A. The plasmids produce an N-terminal hexahistidine-tagged protein with a tobacco etch virus (TEV) protease cleavage site. Site-directed mutants D54C, E59C, R86C, S93C, N115C, E139C, E197C, and E273C were generated using the *Quikchange* protocol (Stratagene) after first preparing a template where three endogenous cysteine residues (at positions 48, 108, and 171) were changed to serines. Mutagenesis primers are detailed in Table S1B. The integrity of all constructs was verified by sequencing.

### Recombinant Protein Production

For isolation of EssB-N, EssB-C, and EssB-C^Δ^, freshly transformed *E. coli* BL21(DE3) pLysS cells were cultivated at 37°C in 10 ml Luria-Bertani (LB) broth containing 50 μg/ml kanamycin and 15 μg/ml chloramphenicol, which was used as inoculum of the 1 l main culture (grown at 37°C in selective LB containing 1 mM MgCl_2_ and 0.5 mM CaCl_2_ in baffled 5 l Erlenmeyer flasks). The temperature was lowered to 25°C when the cells had reached an optical density of 0.6 at λ = 600 nm and expression was induced with 1 mM isopropyl-β-D-thiogalactopyranoside (IPTG).

Cells were harvested after 14 hr, resuspended in buffer A (50 mM sodium phosphate pH 8.0, 300 mM NaCl, 1 mM dithiothreitol, 10% [w/v] glycerol) with the addition of protease inhibitors (Roche), and the homogenates were centrifuged at 150,000 g for 60 min at 4°C. The resulting supernatants were passed through a 0.45-μm filter, imidazole was added to a concentration of 25 mM, and the sample was loaded on a 5-ml HisTrap HP column (GE Healthcare). After a four-column volume (CV) washing step in the same buffer, the recombinant proteins were eluted applying a linear imidazole gradient (25–250 mM over 18 CV). The buffer was exchanged using a spin concentrator (Sartorius) to buffer B (50 mM Tris-HCl pH 7.8, 2 mM dithiothreitol, 10% [w/v] glycerol). After a 14-hr incubation with His-tagged TEV protease (4°C, molar ratio of 1:20 TEV:recombinant proteins), the samples were passed through a HisTrap HP column equilibrated with buffer A to remove the protease, cleaved peptide, and noncleaved material. The cleaved products retain a Gly-Ala-Ser sequence at the N terminus. Fractions containing the target protein were collected, concentrated, and passed through a size exclusion chromatography column (HR 16/60, Superdex75 prep grade, GE Healthcare, CV = 120 ml) equilibrated with buffer C (10 mM sodium phosphate pH 7.8, 20 mM NaCl, 0.5 mM *tris*[2-carboxyethyl]phosphine hydrochloride). The size exclusion chromatography columns had previously been calibrated with molecular mass standards (thyroglobulin, 670 kDa; γ-globulin, 158 kDa; serum albumin, 67 kDa; ovalbumin, 44 kDa; myoglobin, 17 kDa; vitamin B_12_, 1 kDa). SeMet-substituted proteins were obtained using a metabolic inhibition protocol and purified following the protocols just outlined. The mass of the recombinant proteins and the incorporation of SeMet were monitored by matrix-assisted laser-desorption/ionization time-of-flight mass spectrometry (MALDI-TOF-MS) analysis performed at the University of Dundee “Fingerprints” Proteomics Facility using an Applied Biosystems Voyager DE-STR spectrometer.

Preparation of the membrane-bound EssB and EssB^Δ^ followed the same protocol except that LEMO21(DE3) cells (Wagner et al., 2008) were used, the culture medium contained 100 μM L-rhamnose, and gene expression was induced with 400 μM IPTG. Membranes were isolated by centrifugation at 150,000 g for 60 min at 4°C, resuspended in buffer A, and solubilized with *n*-dodecyl-β-D-maltoside (DDM) at a protein-to-detergent mass ratio of 1:3 for 2 hr at 20°C. Nonsolubilized material was removed by centrifugation and the resulting supernatant was purified essentially as described above except that all buffers were supplemented with 0.02% (w/V) DDM and 1 μg/ml 1,2-dioleoyl-*sn*-glycero-3-phosphocholine (DOPC; a kind gift from Lipoid AG, Steinhausen, Switzerland), and an HR 30/100 GL Superdex200 column (CV = 24 ml, GE Healthcare) was used for size exclusion chromatography. Where applicable, DDM was exchanged for 0.1% (w/V) DHPC (1,2-diheptanoyl-sn-glycero-3-phosphocholine) during gel filtration.

### Blue Native Gel Electrophoresis and Analytical Size Exclusion Chromatography of EssB-N

Linear 4%–16% gradient Native Bis-Tris gels (Novex, Life Technologies) were run and destained according to the manufacturer’s protocol. Samples comprising 3 μg of purified EssB-N were loaded. Apoferritin (480 kDa), B-phycoerythrin (242 kDa), lactate dehydrogenase (146 kDa), BSA (66 kDa), and soya bean trypsin inhibitor (20 kDa) were used as standard proteins.

To analyze the stability of the EssB-N dimer by size exclusion chromatography in different buffer systems, EssB-N (2 mg) was buffer exchanged to the test buffer containing 0.5% TCEP and 10% (w/v) glycerol ([a] 10 mM Tris-HCl pH 7.8, 20 mM NaCl; [b] 10 mM Tris-HCl pH 7.8, 20 mM NaCl, 5 mM ethylenediaminetetraacetic acid (EDTA); [c] 100 mM citrate pH 5.5, 300 mM NaCl; [d] 100 mM phosphate pH 7.8, 300 mM NaCl; and [e] 50 mM Bis-Tris pH 5.5, 300 mM NaCl) using a spin concentrator. After incubating the samples for 30 min at room temperature, they were loaded onto a size exclusion chromatography column (HR 30/100 GL, Superdex75 prep grade, GE Healthcare) equilibrated with the same buffer.

### Crystallization and Structure Determination

Crystals of EssB-N were grown by hanging-drop vapor diffusion at 20°C in 3 μl drops of a 1:1 ratio of protein solution (25 mg/ml EssB-N in 20 mM sodium phosphate pH 7.8, 50 mM NaCl, 0.5 mM TCEP) with the reservoir (0.1 M tri-sodium citrate pH 5.5, 20% [w/v] PEG 3000). Bipyramidal crystals of maximum dimensions 100–200 μm appeared after 4 days. The crystals were picked up in LV-oil (MiTeGen, NY, USA), cooled in a stream of gaseous nitrogen at −173°C, then characterized in-house using a Rigaku 007HF rotating anode X-ray generator coupled to a RAXIS IV^2+^ image plate detector. The crystals display space group *P*4_1_2_1_2 with a single molecule in the asymmetric unit.

EssB-C crystals grew in sitting drops of a vapor diffusion setup at 20°C with 1-μl protein stock solution (20 mg/ml EssB-C in 20 mM sodium phosphate pH 7.8, 50 mM NaCl, 0.5 mM TCEP) and 1-μl reservoir (2.1 M malate pH 7.0). The crystals are tetragonal bipyramidal prisms, of maximum dimensions 300 μM, and diffracted to about 3.2 Å resolution. Two molecules constitute the asymmetric unit with a solvent content of approximately 80%. The combination of a large tetragonal unit cell (Table 1), high solvent content, and a flexible C terminus likely explains the poor order. EssB-C^Δ^ produced crystals using sitting drop vapor diffusion at 20°C with 1 μl protein stock solution (20 mg/ml EssB-C^Δ^ in 20 mM sodium phosphate pH 7.8, 50 mM NaCl, 0.5 mM TCEP), and 1 μl reservoir (3.0 M malonate pH 7.0, 10 mM ZnCl_2_). The crystals formed clusters of diamond-like plates. Single fragments were isolated using microtools, plunged in liquid nitrogen, then tested in-house. They belong to space group *C*2 with four molecules in the asymmetric unit and a solvent content of approximately 75%.

EssB-N and EssB-C X-ray data were collected on ID29 at the European Synchrotron Radiation Facility (ESRF, Grenoble, France) and EssB-C^Δ^ data at beam line I02 at Diamond Light Source (DLS, Didcot, UK), all with an ADSC Q315r Charged Couple Device detector. The data were processed using XDS (Kabsch, 2010) except for EssB-N native data, which were processed using Mosflm (Battye et al., 2011).

The crystal structures were solved by SAD using CRANK (Ness et al., 2004), as implemented in the CCP4 suite of programs (Winn et al., 2011). EssB-N presents a single molecule per asymmetric unit and the three expected Se sites were located. Later examination revealed a distinct anomalous signal from a chloride ion (interacting with Arg203 and His207) that was not used for phasing. The initial figure-of-merit (FOM) of 0.34–1.85 Å increased to 0.79 after density modification. A model consisting of residues 36–96 and 110–142 was built using ARP/wARP (Perrakis et al., 1999) and resulted in R_work_ and R_free_ of 0.249 and 0.293, respectively. This model was extended to residues 2–210 in the graphics program COOT (Emsley and Cowtan, 2004) and refined with Refmac5 (Murshudov et al., 2011).

High- and low-resolution data sets, collected using different translations of a single native crystal, were merged using SCALA (Evans, 2006). The SeMet model was positioned in the native unit cell using PHASER (McCoy et al., 2007) then refined in Refmac5 with iterative rounds of electron and difference density map inspection, model manipulation in COOT, and the inclusion of water molecules, chloride, sodium ions, and multiple conformers.

The positions of all six Se atoms in the asymmetric unit of EssB-C were identified and provided phases to 3.8 Å using the SAD approach with an overall FOM of 0.57 (after density modification). A model (chain A residues 251–379, chain B residues 258–382) was built into the electron density map followed by several rounds of refinement. This provided a model to establish the phases of a native data set to 3.2 Å by molecular replacement. Refinement following inspection in COOT gave R_work_ and R_free_ values of 0.443 and 0.466, respectively; however, although the core of the structure was well defined, it was not possible to extend the model. However, the lack of ordered structure at the C terminus of this EssB-C model suggested that a truncation might improve the diffraction order; hence, EssB-C^Δ^ was prepared. This change did indeed result in improved diffraction and a Se-SAD phasing experiment produced an excellent electron density map to 2.9 Å based on 12 Se positions with a FOM of 0.47 (that increased to 0.66 after density modification). All four polypeptides in the asymmetric unit were constructed using BUCCANEER (Cowtan, 2006) and served as a search model to phase native data to 2.4 Å by molecular replacement with PHASER. Rounds of model adjustment using COOT, interspersed with Refmac5 calculations, the addition and refinement of water molecules and components of the crystallization mixture (sodium ions, malonate and glycerol), and inclusion of multiple conformers completed the refinement.

MOLPROBITY (Lovell et al., 2003) was used to monitor model geometry and included a Ramachandran plot analysis during refinements along with the validation tools within COOT. Figures were prepared using PyMOL (Delano, 2002). The DALI server was used to search the PDB for structural homologs and structural superpositions were performed using DALILITE (Holm and Park, 2000). Multiple sequence alignments were calculated using CLUSTALW2 (Larkin et al., 2007) and edited using ALINE (Bond and Schüttelkopf, 2009). The conservation of amino acid sequence was investigated using CONSURF (Landau et al., 2005), noting that EssB-ortholog sequences with identities less than 20% were excluded.

### Computational Structure Prediction

The EssB TM-segment was predicted using TMHMM v2.0 (Krogh et al., 2001). Constructs were generated, in part guided by the *Phyre* fold recognition server (Kelley and Sternberg, 2009).

### Spin-Labeling and EPR Sample Preparation

The cysteine EssB-N and EssB^Δ^ mutant proteins were purified as described above, but with the omission of the thiol-reducing agent TCEP in buffer C. Samples were spectrophotometrically quantified at λ = 280 nm and immediately mixed with a 10-fold molar excess of (1-oxyl-2,2,5,5-tetramethylpyrroline-3-methyl) methanethiosulfonate (MTSSL) from a 10 mg/ml stock solution in dimethylformamide and incubated for 14 hr at 8°C. Labeling was verified by monitoring the 186-Da mass-shift by MALDI-TOF MS analysis.

The spin-labeled samples were buffer exchanged into 20 mM Tris-HCl pH 7.8, 100 mM NaCl (for EssB^Δ^ supplemented with 0.04% DDM and 2 μg/ml DOPC) in D_2_O, and then were diluted with an equal volume of D_8_ glycerol to generate a 100-μl PELDOR sample at a concentration of 200 μM. The samples were transferred into clear, fused quartz EPR tubes and stored at −20°C, and cooled in liquid nitrogen immediately prior to use.

### EPR Data Collection and Data Analysis

PELDOR experiments were carried out using a Bruker ELEXSYS E580 spectrometer operating at X-band with a dielectric ring resonator and a Bruker 400U second microwave source unit. Measurements were made on samples at −233°C with an overcoupled resonator giving a Q-factor of approximately 100. The video bandwidth was set to 20 MHz. A four pulse, dead-time free PELDOR sequence was used, with the pump pulse frequency positioned at the center of the nitroxide spectrum. The frequency of the observer pulses was increased by 80 MHz relative to the pump position. The observer sequence used a 32-ns π-pulse; the pump π-pulse was typically 16 ns. The experiment shot repetition time was 4 ms, and the shots per point were 50. The number of data points and scans used varied for each sample to provide a suitable signal-to-noise ratio. PELDOR data were analyzed using the DeerAnalysis 2011 package (Jeschke et al., 2006). In brief, the time traces of the dipolar coupling evolution data were corrected for background echo decay using a homogeneous three-dimensional spin distribution. The starting time (which corresponds to the zero time of the dipolar evolution data) was optimized to give the best-fit Pake pattern in the Fourier transformed data and the lowest rmsd background fit. Tikhonov regularization was then used to simulate time trace data that gave rise to distance distributions, P(r), of peak width depending on the regularization factor, alpha. The alpha term used was judged by reference to a calculated L-curve. The L-curve is a parametric plot that compares smoothness of the distance distribution to the mean-square-deviation.

### Docking Using Restrained Molecular Dynamics

Spin label positions and conformer distributions were calculated using MtsslWizard (Hagelueken et al., 2012). Multiple nitroxide positions and distributions were incorporated into the monomer structure and the dimer models were refined by rigid body dynamics using Xplor-NIH (Schwieters et al., 2003) at 227**°**C, with restraints taken from PELDOR-derived distance distributions. Multiple rounds of refinement were carried out with starting structures in which pairs of monomers, with random orientations, were separated by approximately 50 Å. Final structures were selected at the lowest distance violation. Distance distributions for the final model were calculated using MtsslWizard running within PyMOL.

## Figures and Tables

**Table 1:** Crystallographic Statistics for EssB-N

Data Set	SeMet (peak)	Native
Spacegroup	*P*4_1_2_1_2	*P*4_1_2_1_2
Wavelength (Å)	0.9793	0.9801
Resolution (Å)	44.74–1.85 (1.95–1.85)^a^	44.61–1.70 (1.79–1.70)
Unit cell (Å)	74.7 74.7 84.3	74.6 74.6 83.5
Unique reflections	20,759 (2,661)	26,751(3,814)
Completeness (%)	97.7 (84.8)	100 (100)
<*I/σ(I)* >	22.7 (1.6)	24.4 (4.3)
Multiplicity	13.3 (7.8)	9.6 (7.1)
R_merge_^b^	0.066 (1.077)	0.053 (0.416)

**Refined Model**		

Resolution range (Å)		44.61–1.70
R_work_^c^		20.3
R_free_^d^		25.8
Protein residues		209
Other components		300 waters, 1 Na^+^, 1 Cl^−^

**Rmsd from Ideal Geometry**		

Bond lengths (Å)		0.009
Bond angles (^°^)		1.211

**Thermal Parameter (B) Values (Å2)**		

From Wilson plot		29.9
Mean *B* over all atoms		24.1
Ramachandran favored/allowed/outliers (%)		97.1/2.9/0

a Values in parentheses refer to the highest resolution bin.

b R_merge_ = ∑*h∑i||*(*h,i*) − < I(*h*) > ∑*h∑i* I(*h,i*).

c R_work_ = ∑*hkl*||*F*_*o*_|−|*F*_*c*_||/∑*|F*_*o*_*|,* where *F*_*o*_ is the observed structure-factor amplitude and the *F*_*c*_ is the structure-factor amplitude calculated from the model.

d R_free_ is the same as R_work_ except only calculated using a subset, 5%, of the data that are not included in any refinement calculations.

**Table 2:** Crystallographic Statistics for EssB-C and EssB-C^Δ^

Data Set	EssB-C SeMet (Peak)	EssB-C (Native)	EssB-C^Δ^ SeMet (Peak)	EssB-C^Δ^ (Native)
Construct	Ala241-Lys428	Ala241-Lys428	Ala243-Gln397	Ala243-Gln397
Spacegroup	*P*4_3_2_1_2	*P*4_3_2_1_2	*C*2	*C*2
Wavelength (Å)	0.9792	1.0070	0.9794	0.9801
Resolution (Å)	48.06–3.80 (4.01–3.80)^a^	48.17–3.20 (3.27–3.20)	29.46–2.87 (3.03–2.87)	64.65–2.40 (2.53–2.40)
Unit cell (Å)	94.9 94.9 206.7	95.6 95.6 206.0	133.7 110.4 97.3 β = 103.3°	132.9 110.6 97.4 β = 103.3°
Unique reflections	9,901 (1,399)	16,402 (2,334)	31,181 (4,220)	52,114 (7,558)
Completeness (%)	99.9 (100)	99.4 (100)	98.8 (92.5)	97.4 (97.4)
<I/σ(I) >	20.5 (3.5)	26.6 (2.2)	14.3 (2.8)	11.7 (3.0)
Redundancy	8.9 (9.3)	8.2 (8.5)	5.5 (4.6)	4.6 (4.7)
R_merge_^b^	0.051 (0.525)	0.048 (0.698)	0.070 (0.470)	0.069 (0.438)

**Refined Model**				

Resolution range (Å)		55.31–2.40		
No. reflections		49,452		
R_work_^c^		18.6		
R_free_^d^		22.1		
Protein residues (chain)		148 (A), 150 (B), 148 (C), 150 (D)		
Other components		275 waters, 5 Na^+^, 3 Cl^−^, 3 malonates, 1 glycerol		

**Rmsd from Ideal Geometry**				

Bond lengths (Å)		0.011		
Bond angles (^°^)		1.20		

**B Values (Å**^**2**^**)**				

From Wilson plot		56.6		
Mean *B* over all atoms		43.9		
Ramachandran favored/allowed/outliers (%)		98.3/1.5/0.2		

a Values in parentheses refer to the highest resolution bin.

b R_merge_ = ∑*h∑i||*(*h,i*) − < I(*h*) > ∑*h∑i* I(*h,i*).

c R_work_ = ∑*hkl*||*F*_*o*_|−|*F*_*c*_||/∑*|F*_*o*_*|,* where *F*_*o*_ is the observed structure-factor amplitude and the *F*_*c*_ is the structure-factor amplitude calculated from the model.

d R_free_ is the same as R_work_ except calculated using a subset, 5%, of the data that are not included in any refinement calculations.

**Table 3:** Summary of PELDOR Results

Spin Label Site	Simulated Distance Distributions from Final Model		Experimental Distance Distributions	
	Modal Distances	Width at Half-Height	Modal Distances	Width at Half-Height
D54C	53	8.3	55	4.4
E59C	66	3.8	65	4.6
E139C	58	5.9	58	7.0
E197C	39	6.0	38	6.2
E273C	43	6.1	44	6.2

Comparison of distances and distributions (Å) using the EssB-N dimer model and, for E273C, the EssB-C^Δ^ X-ray structure.

## Accession Numbers

The coordinates and structure-factor data for EssB-N and EssB-C^Δ^ are deposited with the PDB under the accession codes 4ANO and 2YNQ, respectively.

## Supplemental Information

Document S1. Figures S1–S3 and Table S1

Movie S1. Predicted Architecture of EssB with Spin-Labeling Positions, Related to Figure 2 Schematic EssB model in cartoon representation shows positions of MTSSL labels. Ensembles of possible MTSSL nitrogen positions on the five mutant positions are shown as color-coded mesh for clarity (D54C in red, E59C in green, E139C in dark blue, E197C in orange, and E273C in light blue). The model is assembled with the EssB-C dimer and modeled TM-segment (yellow) extended down through the membrane toward the docked cytoplasmic EssB-N fragments.

## References

[bib1] Abdallah A.M., Gey van Pittius N.C., Champion P.A.D., Cox J., Luirink J., Vandenbroucke-Grauls C.M.J.E., Appelmelk B.J., Bitter W. (2007). Type VII secretion—mycobacteria show the way. Nat. Rev. Microbiol..

[bib2] Battye T.G., Kontogiannis L., Johnson O., Powell H.R., Leslie A.G. (2011). iMOSFLM: a new graphical interface for diffraction-image processing with MOSFLM. Acta Crystallogr. D Biol. Crystallogr..

[bib3] Bond C.S., Schüttelkopf A.W. (2009). ALINE: a WYSIWYG protein-sequence alignment editor for publication-quality alignments. Acta Crystallogr. D Biol. Crystallogr..

[bib4] Burts M.L., Williams W.A., DeBord K., Missiakas D.M. (2005). EsxA and EsxB are secreted by an ESAT-6-like system that is required for the pathogenesis of *Staphylococcus aureus* infections. Proc. Natl. Acad. Sci. USA.

[bib5] Chen Y.H., Anderson M., Hendrickx A.P., Missiakas D. (2012). Characterization of EssB, a protein required for secretion of ESAT-6 like proteins in *Staphylococcus aureus*. BMC Microbiol..

[bib6] Cowtan K. (2006). The Buccaneer software for automated model building. 1. Tracing protein chains. Acta Crystallogr. D Biol. Crystallogr..

[bib7] Delano W.L. (2002). The PyMOL Molecular Graphics System.

[bib8] Emsley P., Cowtan K. (2004). Coot: model-building tools for molecular graphics. Acta Crystallogr. D Biol. Crystallogr..

[bib9] Evans P. (2006). Scaling and assessment of data quality. Acta Crystallogr. D Biol. Crystallogr..

[bib10] Hagelueken G., Ward R., Naismith J.H., Schiemann O. (2012). MtsslWizard: in silico spin-Labeling and generation of distance distributions in PyMOL. Appl. Magn. Reson..

[bib11] Holm L., Park J. (2000). DaliLite workbench for protein structure comparison. Bioinformatics.

[bib12] Houben E.N., Bestebroer J., Ummels R., Wilson L., Piersma S.R., Jiménez C.R., Ottenhoff T.H., Luirink J., Bitter W. (2012). Composition of the type VII secretion system membrane complex. Mol. Microbiol..

[bib13] Jeschke G., Koch A., Jonas U., Godt A. (2002). Direct conversion of EPR dipolar time evolution data to distance distributions. J. Magn. Reson..

[bib14] Jeschke G., Chechik V., Ionita P., Godt A. (2006). DeerAnalysis2006 - A comprehensive software package for analyzing pulsed ELDOR data. Appl. Magn. Reson..

[bib15] Kabsch W. (2010). XDS. Acta Crystallogr. D Biol. Crystallogr..

[bib16] Kelley L.A., Sternberg M.J.E. (2009). Protein structure prediction on the Web: a case study using the Phyre server. Nat. Protoc..

[bib17] Krissinel E., Henrick K. (2007). Inference of macromolecular assemblies from crystalline state. J. Mol. Biol..

[bib18] Krogh A., Larsson B., von Heijne G., Sonnhammer E.L. (2001). Predicting transmembrane protein topology with a hidden Markov model: application to complete genomes. J. Mol. Biol..

[bib19] Landau M., Mayrose I., Rosenberg Y., Glaser F., Martz E., Pupko T., Ben-Tal N. (2005). ConSurf 2005: the projection of evolutionary conservation scores of residues on protein structures. Nucleic Acids Res..

[bib20] Larkin M.A., Blackshields G., Brown N.P., Chenna R., McGettigan P.A., McWilliam H., Valentin F., Wallace I.M., Wilm A., Lopez R. (2007). Clustal W and Clustal X version 2.0. Bioinformatics.

[bib21] Levinson N.M., Kuchment O., Shen K., Young M.A., Koldobskiy M., Karplus M., Cole P.A., Kuriyan J. (2006). A Src-like inactive conformation in the abl tyrosine kinase domain. PLoS Biol..

[bib22] Lewis K.N., Liao R., Guinn K.M., Hickey M.J., Smith S., Behr M.A., Sherman D.R. (2003). Deletion of RD1 from *Mycobacterium tuberculosis* mimics bacille Calmette-Guérin attenuation. J. Infect. Dis..

[bib23] Liang X., Van Doren S.R. (2008). Mechanistic insights into phosphoprotein-binding FHA domains. Acc. Chem. Res..

[bib24] Lovell S.C., Davis I.W., Arendall W.B., de Bakker P.I.W., Word J.M., Prisant M.G., Richardson J.S., Richardson D.C. (2003). Structure validation by Calpha geometry: phi,psi and Cbeta deviation. Proteins.

[bib25] McCoy A.J., Grosse-Kunstleve R.W., Adams P.D., Winn M.D., Storoni L.C., Read R.J. (2007). Phaser crystallographic software. J. Appl. Cryst..

[bib26] McDowell M.A., Johnson S., Deane J.E., Cheung M., Roehrich A.D., Blocker A.J., McDonnell J.M., Lea S.M. (2011). Structural and functional studies on the N-terminal domain of the *Shigella* type III secretion protein MxiG. J. Biol. Chem..

[bib27] Milov A.D., Salikhov K., Shirov M. (1981). Use of the double resonance in electron spin echo method for the study of paramagnetic center spatial distribution in solids. Fizika Tverdogo Tela.

[bib28] Murshudov G.N., Skubák P., Lebedev A.A., Pannu N.S., Steiner R.A., Nicholls R.A., Winn M.D., Long F., Vagin A.A. (2011). REFMAC5 for the refinement of macromolecular crystal structures. Acta Crystallogr. D Biol. Crystallogr..

[bib29] Ness S.R., de Graaff R.A., Abrahams J.P., Pannu N.S. (2004). CRANK: new methods for automated macromolecular crystal structure solution. Structure.

[bib30] Pallen M.J. (2002). The ESAT-6/WXG100 superfamily — and a new Gram-positive secretion system?. Trends Microbiol..

[bib31] Pannier M., Veit S., Godt A., Jeschke G., Spiess H.W. (2000). Dead-time free measurement of dipole-dipole interactions between electron spins. J. Magn. Reson..

[bib32] Pereira S.F.F., Goss L., Dworkin J. (2011). Eukaryote-like serine/threonine kinases and phosphatases in bacteria. Microbiol. Mol. Biol. Rev..

[bib33] Perrakis A., Morris R., Lamzin V.S. (1999). Automated protein model building combined with iterative structure refinement. Nat. Struct. Biol..

[bib34] Schiemann O., Prisner T.F. (2007). Long-range distance determinations in biomacromolecules by EPR spectroscopy. Q. Rev. Biophys..

[bib35] Schwieters C.D., Kuszewski J.J., Tjandra N., Clore G.M. (2003). The Xplor-NIH NMR molecular structure determination package. J. Magn. Reson..

[bib36] Tanaka Y., Kuroda M., Yasutake Y., Yao M., Tsumoto K., Watanabe N., Ohta T., Tanaka I. (2007). Crystal structure analysis reveals a novel forkhead-associated domain of ESAT-6 secretion system C protein in *Staphylococcus aureus*. Proteins.

[bib37] Wagner S., Klepsch M.M., Schlegel S., Appel A., Draheim R., Tarry M., Högbom M., van Wijk K.J., Slotboom D.J., Persson J.O., de Gier J.W. (2008). Tuning *Escherichia coli* for membrane protein overexpression. Proc. Natl. Acad. Sci. USA.

[bib38] Winn M.D., Ballard C.C., Cowtan K.D., Dodson E.J., Emsley P., Evans P.R., Keegan R.M., Krissinel E.B., Leslie A.G.W., McCoy A. (2011). Overview of the CCP4 suite and current developments. Acta Crystallogr. D Biol. Crystallogr..

[bib39] Zoltner M., Fyfe P.K., Palmer T., Hunter W.N. (2013). Characterization of Staphylococcus aureus EssB, an integral membrane component of the Type VII secretion system: atomic resolution crystal structure of the cytoplasmic segment. Biochem. J..

