# Supplementary material for: The Architecture of EssB, an Integral Membrane Component of the Type VII Secretion System
Source: Structure. 2013 Apr 2;21(4):595–603. doi: 10.1016/j.str.2013.02.007 (PMC3694306; doi:10.1016/j.str.2013.02.007)
Supplement: Document S1. Figures S1–S3 and Table S1 [file mmc1.pdf]

Structure, Volume *21*

Supplemental Information

The Architecture of EssB, an Integral Membrane Component of the Type VII Secretion System

Martin Zoltner, David G. Norman, Paul K. Fyfe, Hassane El Mkami, Tracy Palmer, and William N. Hunter

<u>Inventory of Supplemental Information</u>

Figure S1. Stereo views and structural comparison of *S.aureus* and *G.thermodenitrificans* EssB-N (related to Figure 1).

Figure S2. Dimer architecture and EssB-N dimer stability (related to Figure 2).

Figure S3. Spectroscopic and sequence data

Movie S1. Predicted architecture of EssB with spin labelling positions (related to Figure 2).

Table S1.A Cloning primers (related to EXPERIMENTAL)

Table S1.B Mutagenesis primers (related to EXPERIMENTAL)

Figure S1. Stereo views and structural comparison of *S.aureus* and *G.thermodenitrificans* EssB-N (related to Figure 1).

Stereo view of Cα trace of EssB-N (A) and EssB-C^{Δ} (B). The chains are colored by secondary structure elements (black = loop, red = helix, blue = strand) and residue numbers are indicated at every tenth C-alpha (shown as spheres). Malonate and glycerol are drawn as grey sticks and sodium and chloride ions as bronze and grey colored spheres, respectively.

(C) Structural comparison of *S.aureus* and *G.thermodenitrificans* EssB-N. Rigid body superimposition of *S.aureus* EssB-N (blue, PDB code 4ann, Zoltner et al., 2013) and *G.thermodenitrificans* EssB-N (green, PDB) over 170 Cα-atoms (see RESULTS AND DISCUSSION).

Figure S2. Dimer architecture and EssB-N dimer stability (related to Figure 2).

(A) BN-PAGE analysis of purified EssB-N. EssB-N (left lane) migrates as dimer with an apparent molecular weight of 45 kDa, derived by comparison with molecular mass standards (right lane, masses given in kDa and a calibration curve are indicated; see description in the main text).

(B) Overlay of size exclusion chromatography chromatograms for EssB-N. 2 mg protein was loaded on a *Superdex75* (GE healthcare) column equilibrated with 10 mM Tris pH 7.8, 20 mM NaCl (black) or 50mM Citrate pH 5.5, 300mM NaCl (grey). Samples were pre-equilibrated in the running buffer before loading. Peaks are labeled with apparent molecular weights (see EXPERIMENTAL).

(C) Conservation surface mapping onto EssB-C^{Δ}. Topside view of the EssB-C^{Δ} dimer. One subunit is depicted with ribbon format and the other with conservation scores (based on the alignment shown in Supplementary Figure S8) mapped on the surface. The continuous conservation scores are normalized and partitioned into 9 discrete conservation bins (darkest pink indicates the most conserved), reflecting the relative degree of conservation of each amino acid position (see EXPERIMENTAL).

(D) Predicted architecture of EssB with spin labelling positions.Schematic EssB-N model in cartoon representation showing positions of MTSSL-label pairs and the resulting distances. MTSSL nitrogen positions representing the maxima of the corresponding simulated distance distributions on the four mutant positions are shown as spheres (D54C in red, E59C in green, E139C in dark blue, E197C in orange). Experimental distances (determined in detergent solubilized EssB-C^{Δ}) for each label pair are indicated (see Table 3, Supplementary Figures S6 and S7)

Figure S3. Spectroscopic and sequence data

(A, B) PELDOR Data and Distance Distribution: (A) Dipolar oscillation (red) and corrected dipolar oscillation (black) (left) and Tikhonov derived distance distribution (right, red). The synthesized distance distribution derived from EssB-C$^{\Delta}$ crystal structure (right). (B) Graphs showing Tikhonov derived distance distribution for positions on the cytoplasmic portion EssB-N (D54C, E59C, E138C and E197C) investigated on detergent solubilized EssB$^{\Delta}$. The distance distribution for D54C (top left) was additionally investigated on the isolated soluble fragment EssB-N (dashed line).

(C, D) PELDOR raw data: Graphs showing dipolar oscillations for all positions on the cytoplasmic portion EssB-N investigated on detergent solubilized EssB. (C) Four label positions (D54C, E59C, E139C and E197C) giving clear oscillations and distance data that was used for generating the dimer model. (D) Oscillation free data was recorded for three label positions (R86C, S93C, N115C) occluded by the interface of the dimer model

(E) Experimental and simulated distance distributions. Tikhonov derived distance distribution and distance distributions simulated from the final model.

(F) Multiple sequence alignment of EssB orthologues. Secondary structure elements for *S.aureus* EssB (orange, top) and *G.thermodenitrificans* EssB (green, bottom) and the predicted transmembrane span (TM) are indicated. Predicted secondary structure elements are drawn in grey. The selected EssB sequences are titled with *UniProt*-identifier and species abbreviation (Lin=*Listeria innocua*, Lse=*Listeria seeligeri*, Lgr=*Listeria grayi*, Bth=*Bacillus thuringiensis*, Bce= *Bacillus cereus*, Bps= *Bacillus pseudomycoides*, Bmy=*Bacillus mycoides*, Oih=*Oceanobacillus iheyensis*, Sor=*Streptococcus oralis*, Sag=*Streptococcus agalactiae*, Sep=*Staphylococcus epidermis*, Slu= *Staphylococcus lugdunensis*, Sau=*Staphylococcus aureus*, Pmi=*Parvimonas micra*, Gha=*Gemella haemolysans*, Sin=*Streptococcus infantis*, Cmo=*Catonella morbi*, Csp=*Clostridium spiroforme*, Cne= *Clostridium nexile*, Esa=*Eubacterium saburreum*, Lra= *Lachnospiraceae bacterium*, Cac= *Clostridium acetobutylicum*, Efa= *Enterococcus faecalis*, Bsu=*Bacillus subtilis*, Bam=*Bacillus amyloliquefaciens*, Bli= *Bacillus licheniformis*, Bme= *Bacillus megaterium*, Gtg=*Geobacillus thermoglucosidans*, Gth=*Geobacillus thermodenitrificans*)

(G, H) Surface representation of the EssB-N model: (G) Van der Waals surface: One subunit is depicted with ribbon format and the other has the van der Waals surface

shown and colored according to chemical properties; red acidic, blue basic, white non-polar. Labeled secondary structure elements and regions are discussed in the text.

(H) Conservation surface mapping: one subunit is depicted with ribbon format and the other with conservation scores (based on the alignment shown in Supplementary Figure S8) mapped on the surface. The continuous conservation scores are normalized and partitioned into 9 discrete conservation bins (darkest pink indicates the most conserved), reflecting the relative degree of conservation of each amino acid position (see Experimental).

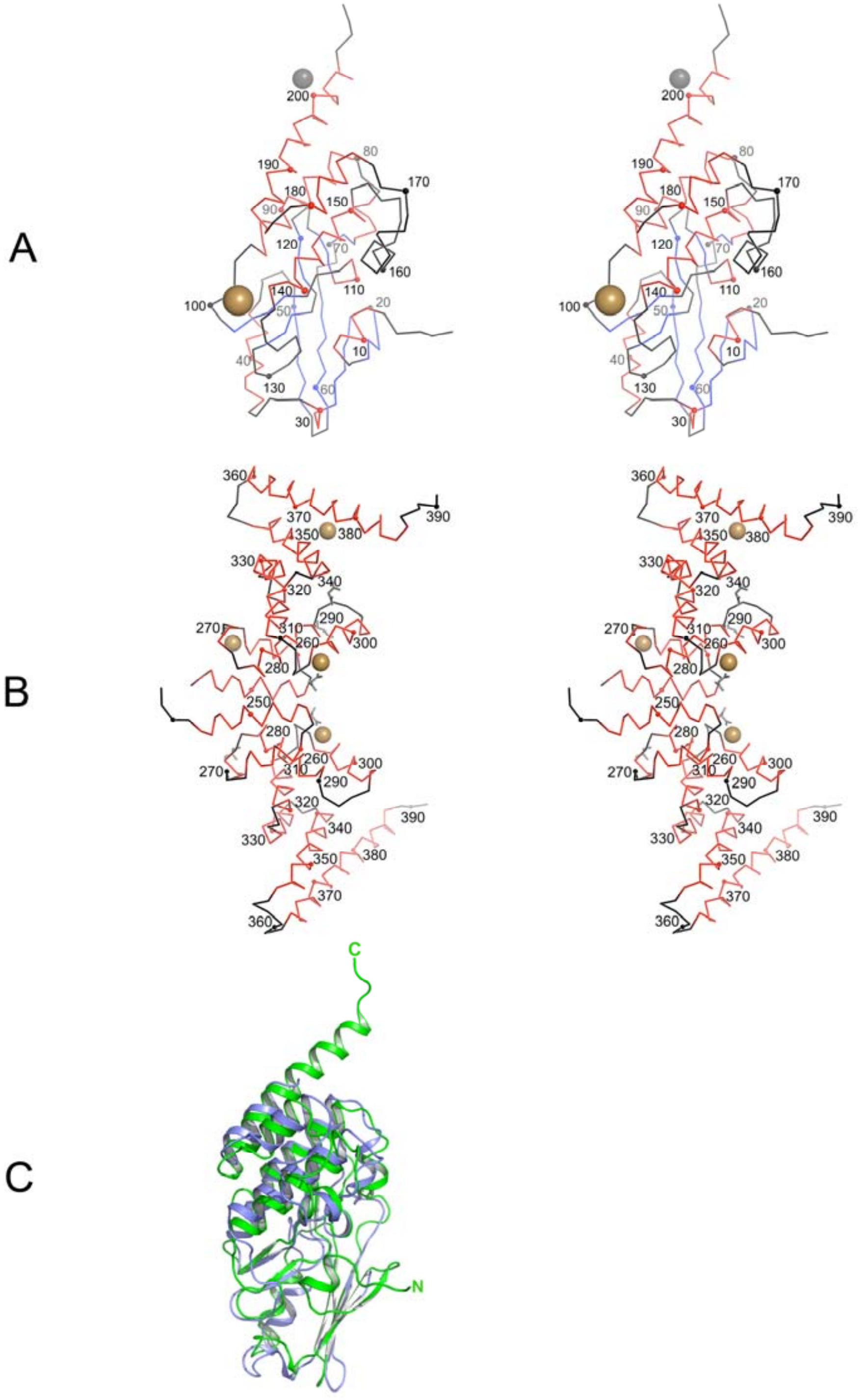

Figure S1. (related to Figure 1).

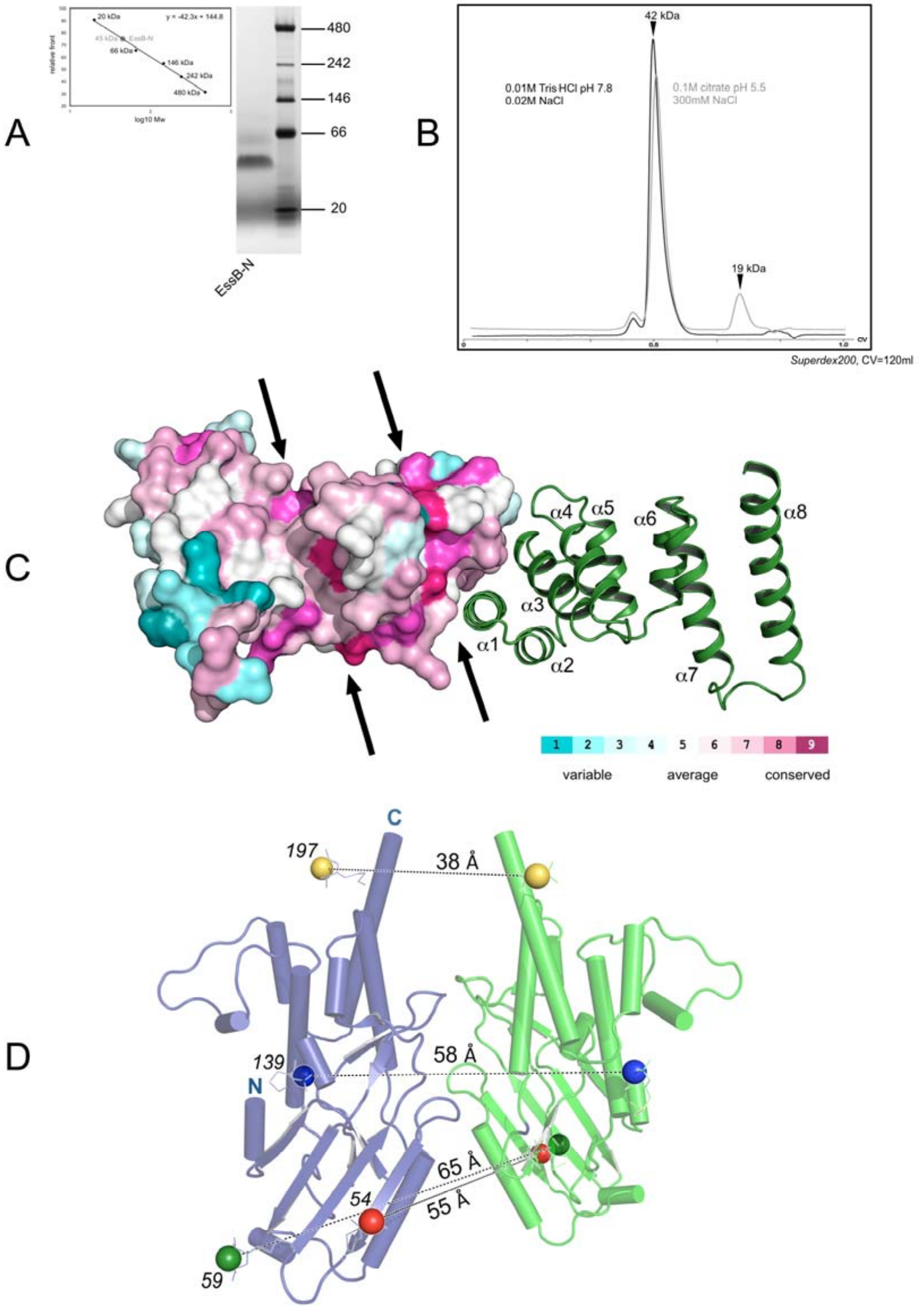

Figure S2. (related to Figure 2).

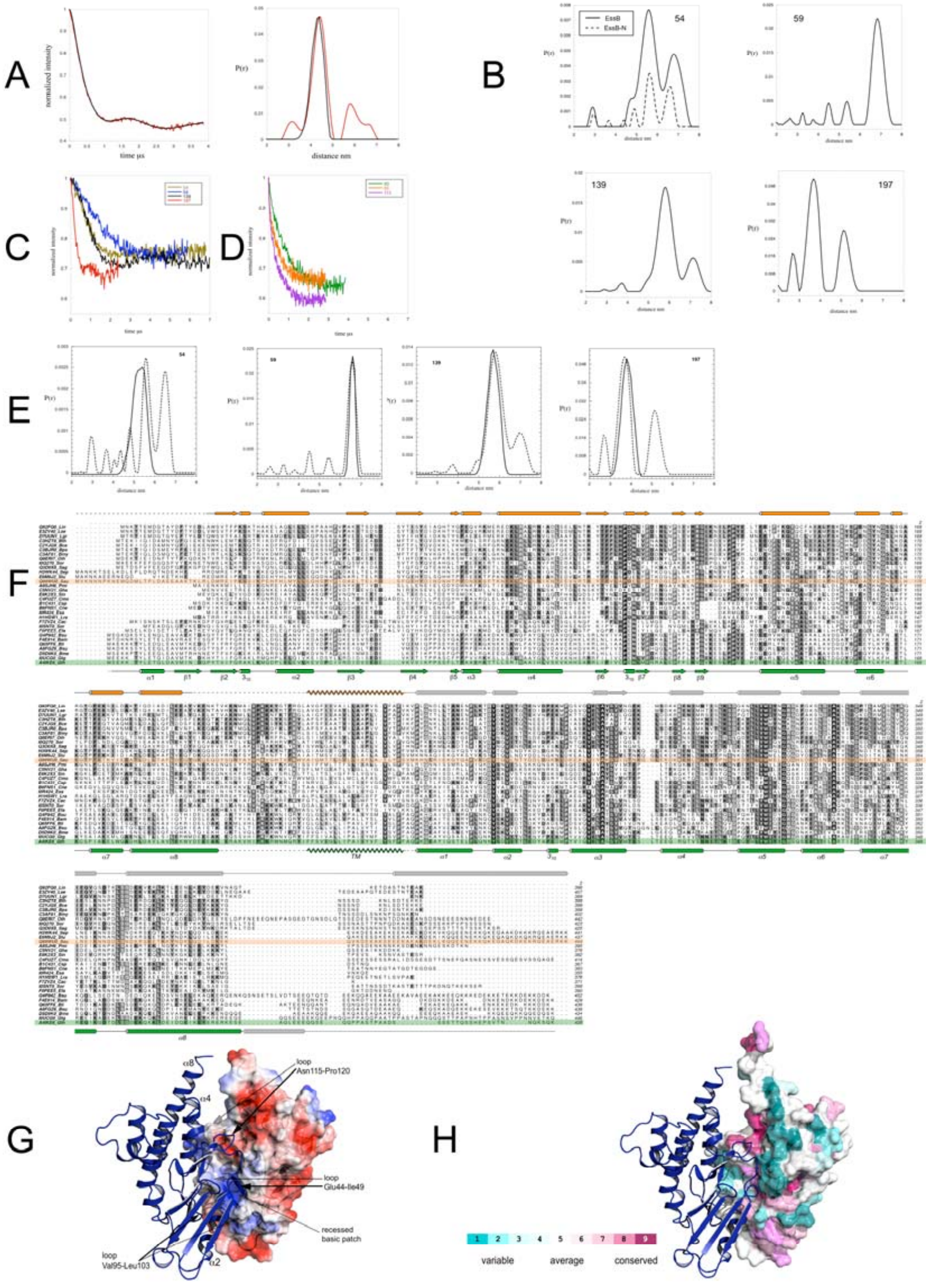
A
normalized intensity
time μs
P(r)
distance nm
B
EssB
EssB-N
54
59
139
197
P(r)
C
normalized intensity
time μs
D
E
F
G
loop
Asn115-Pro120
α8
α4
loop
Glu44-Ile49
recessed
basic patch
loop
Val95-Leu103
α2
H
1
2
3
4
5
6
7
8
9
variable
average
conserved

Figure S3.

Table S1.A Cloning primers

construct	Primer pair
EssB	5'- GCGCGTCGACAAGCGAAAAAAAAACCTATCTGGAAACCCAG -3'<br>5'- GCGCCTCGAGTCATTTCTGGCTTTTCTGGTTGTTGG -3'
EssB$^{\Delta}$	5'- GCGCGTCGACAAGCGAAAAAAAAACCTATCTGGAAACCCAG -3'<br>5'- GCGCGCCTCGAGTCACTGCTGTTCGCTCTGCTGTTCTTC -3'
EssB-N	5'- GCGCGTCGACAAGCGAAAAAAAAACCTATCTGGAAACCCAG -3'<br>5'- GCGCGCCTCGAGTCAGTTCCAGCGTTTTTTCGGAATATGC -3'
EssB-C	5'- GCGCGCGTCGACAGCGCAGCCGAAACAGGAAGCG -3'<br>5'- GCGCCTCGAGTCATTTCTGGCTTTTCTGGTTGTTGG -3'
EssB-C$^{\Delta}$	5'- GCGCGCGTCGACAGCGCAGCCGAAACAGGAAGCG -3'<br>5'- GCGCGCCTCGAGTCACTGCTGTTCGCTCTGCTGTTCTTC -3'

Table S1.B Mutagenesis primers

Mutation	Mutagenesis-primer pair
C48S	5'-GAAAGAAGTGGATCCGAGCATTGTGCGCGATATTG-3'<br>5'-CAATATCGCGCACAATGCTCGGATCCACTTCTTTC-3'
C108S	5'-CGCCTGATTTTTATTGTGAGCCCGGAAAACCTGATG-3'<br>5'-CATCAGGTTTTCCGGGCTCACAATAAAAATCAGGCG-3'
C171S	5'-CATGAAACCCTGAAAAGCAGCCCGATTGCGAAAG-3'<br>5'-CTTTCGCAATCGGGCTGCTTTTCAGGGTTTCATG-3'
D54C	5'-CCGTGCATTGTGCGCGATATTTGCGTGAGCGAAGATGAAGT GAAAGTG -3'<br>5'-CACTTTCACTTCATCTTCGCTCACGCAAATATCGCGCACAAT GCACGG-3'
E59C	5'-CGATATTGATGTGAGCGAAGATTGCGTGAAAGTGGTGATTAAAC C-3'<br>5'-GGTTTAATCACCACTTTCACGCAATCTTCGCTCACATCAATATCG-3'
R86C	5'-CACCCTGCTGAGCCGCATTTGCGCGGCGATTCATCTGGTG -3'<br>5'-CACCAGATGAATCGCCGCGCAAATGCGGCTCAGCAGGGTG-3'
S93C	5'-GCGGCGATTCATCTGGTGTGCAAAGTGAAACATCATAGCG-3'<br>5'-CGCTATGATGTTTCACTTTGCACACCAGATGAATCGCCGC-3'
N115C	5'-CCGGAAAACCTGATGTTTTGCCGCGCGCTGGAACCG-3'<br>5'-CGGTTCCAGCGCGCGGCAAAACATCAGGTTTTCCGG-3'
E139C	5'-CCGGATGAATGGGATGATTGCCGCCTGCTGCGCGAAGTG-3'<br>5'-CACTTCGCGCAGCAGGCGGCAATCATCCCATTCATCCGG-3'
E197C	5'- GCTGGAAAAATGGGTGGAT**TGT**GAAGAAGCGAAAGAACGC-3'<br>5'- GCGTTCTTTCGCTTCTTCACAATCCACCCATTTTTCCAGC-3'
E273C	5'CCTGGAAGATTATGCGCCGTGCGATATGCCGTATGTGATTCAG-3'<br>5'-CTGAATCACATACGGCATATCGCACGGCGCATAATCTTCCAGG-3'
